# Drug Shortage and Ethical Issues: Integrating Multidisciplinary Perspectives with a Shared Ethical Framework

**DOI:** 10.3390/pharmacy12050136

**Published:** 2024-09-06

**Authors:** Maya C. Wai

**Affiliations:** College of Pharmacy, Department of Pharmacy Practice, Northwest Regional Campus, University of Arkansas for Medical Sciences, 1125 N. College Ave., Fayetteville, AR 72703, USA; mcwai@uams.edu

**Keywords:** ethics, drug shortages, economics, multidisciplinary

## Abstract

Drug shortages can cause ethical dilemmas when no systematic, equitable allocation, or utilization schema is in place. During the COVID-19 pandemic, an ethical framework outlining moral values was proposed as way to approach allocating limited resources to patients. In addition to an ethical perspective, it is prudent to consider costs. Examining existing economic frameworks and combining them with an ethical perspective may provide a practical, systematic process for decision makers when allocating drugs in short supply. Drug shortages continue to impact multiple areas across different subspecialties of medicine due to multiple factors, including limited manufacturers, regulatory issues, and costs. All of these factors make it difficult to anticipate and manage drug shortages effectively, but developing a combined framework may reduce some of the ethical and equitable ambiguity with regards to patient care.

## 1. Introduction

Drug shortages are a continuous challenge across the entire healthcare system that is multi-causal in nature [1]. Drug shortages are a global problem and are often caused by supply chain issues and bottlenecks across multiple countries and vast multinational networks [2]. Recently, the American Society of Health-System Pharmacists (ASHP) provided possible policy solutions to address the drug shortage crisis, pointing out that currently there are over 300 active drug shortages according to the ASHP and University of Utah drug shortages database [3], an initiative to increase the transparency of drug shortages and provide accurate and up-to-date information for various stakeholders.

Additionally, technical, economic, political, and policy issues appear to be driving continued drug shortages in the U.S. According to ASHP [3]: “the most severe and persistent shortages are driven by economic factors that undermine investment in manufacturing capacity, manufacturing quality, and supply chain reliability”. Among the multiple parties involved, such as manufacturers, intermediaries (e.g., pharmacy benefit managers), patient-facing players (e.g., hospitals), and patients and employers, the issues of politics and policy come to the forefront when policy regulations and legal disputes are added to the mix (e.g., [4]).

The term “multidisciplinary” in this paper refers to the different disciplines of medical researchers, health economists, policymakers, ethicists, physicians, and pharmacists. Medicine and healthcare in general would benefit from inviting all of these disciplines into discussions and the management of drug shortages to advance research, policy, and practice.

## 2. The Literature on Drug Shortages Spans Multiple Medical Subspecialties and Could Be Better Integrated

### 2.1. The Drug Shortage Literature Spans Multiple (Often Disconnected) Medical Subspecialties

In a recent scoping review of the drug shortage literature from 2001 to 2019, Tucker et al. [5] illustrate that the majority of the drug shortage literature has come about starting roughly in 2011, so in the last 10 to 15 years. The drug shortage literature is wide-ranging and spans numerous, somewhat disconnected, subspecialties that publish in their individual field journals.

Chan ([6], p. 702), writing on the issue of drug shortages likely impacting iatrogenic malnutrition, notes that “Drug shortage is hardly a new issue. But it has reached a crisis situation in the past 3 years”. This was stated in 2013, showing that despite the published literature and some media stories, in addition to policy reports and advocacy, the crisis situation continues. Chan argues that in his field journal of *Journal of Parenteral and Enteral Nutrition*, there was little media attention, but that for journals such as *Lancet*, *NEJM*, *BMJ*, or *Science*, and on the topic of cancer, there was much attention. Other examples of drug shortages include the impact on children with cancer (i.e., Mechlorethamine; [7]), saline (e.g., [8,9]), or catheter-related blood stream infections [10], to just highlight a few. Much of the research examining drug shortage issues in various fields is case studies, descriptive in nature, or quasi-experimental.

### 2.2. The Economic Policy Perspective Is Useful, but Needs to Be Integrated with Other Field Perspectives

Tucker et al. ([5], p. 1154) also note that “Few analyses exist on the potential effects of policies to prevent shortages. Other disciplines may be able to add insight to this area, including economists, industrial engineers, and psychologists”. Economists are highly influential in policy research generally, and their methods are focused on forward causal inference (see [11] for a summary of methods used).

The economic approach is standard for much of policy analysis outside of the drug shortage literature. A summary of this perspective is provided by Wosinska [12], who argues that “Different shortages have different causes. To be effective, policy solutions must map to those causes”. Additionally, she notes “Without a strategic approach that recognizes different causes of shortages, we risk implementing expensive fixes that do little to make the U.S. drug supply chains more reliable”. She describes four important questions to consider when implementing policy solutions: 1. Does the policy address the actual cause(s) of the shortages? 2. Can the policy work by itself or does its effectiveness depend on the presence of other policies? 3. Does the policy anticipate and limit potential unintended consequences? 4. Is the policy cost-effective relative to alternatives? However, because policy is often connected to politics and values, the economics perspective could also consider issues such as ethics, instead of focusing primarily on return on investment and cost. Wosinska [12] uses the economic framework to argue that because drug shortages are multi-causal in nature, that solutions must actually address the root causes. Calls to action without consideration of cost may not necessarily be helpful. However, this is where other field perspectives are useful.

## 3. Drug Shortages Create Ethical Dilemmas

Drug shortages create the ethical dilemma of scarce resources, which creates issues ranging from patients not being able to find needed medications at local pharmacies, to doctors not being able to prescribe the optimal medications. According to a survey conducted by ASHP [13], over 99% of respondents (largely pharmacists) reported that drug shortages impacted their work, particularly chemotherapy drugs, and their budgets.

There is literature on how to manage drug shortages (e.g., for oncology [14]), but it remains unclear whether the alternative treatments provided are actually effective [15], or if the decision making in the allocation of resources was performed in the most ethically optimal way, or at the very least based on core principles [16].

The allocation of scarce medical resources became critical during the COVID-19 pandemic. As Emanuel and Persad ([17], p. 1892) argue, “In the past 100 years, no other event or novel technology—not the advent of penicillin, dialysis, organ transplantation, or new genetic therapeutics—has necessitated the allocation of scarce health resources for more people worldwide than the COVID-19 pandemic.” Whether the actual triage decisions regarding scarce medical resources were ethically or optimally conducted is questionable [18]; however, what appears to have emerged is a shared ethical framework aligned with Universal Human Rights which came from multiple global reports on allocating scarce medical resources [17].

### A Shared Ethical Framework Based on Universal Human Rights

Emanuel and Persad [17] distill a core ethical framework which is composed of the following: maximizing benefit and reducing harm, mitigating disadvantage (equity), equal moral concern, reciprocity, and instrumental value. Table 1 shows each of these core aspects and their corresponding definitions.

By combining the ethical values from Emanuel and Persaud with the economic framework in the context of the wider published literature, the combined framework would look like this as a set of questions:

## 4. The Multidisciplinary Ethical Framework for Allocating Scarce Medical Resources

1. Does the framework address the actual cause (s) of the shortages?
2. Can the framework work by itself or does its effectiveness depend on the presence of other policies?
3. Does the framework anticipate and limit potential unintended consequences?
4. Is the framework cost-effective relative to alternatives?
5. Does the framework adequately address ethical issues?
6. What other evidence (e.g., case studies, descriptive findings) should be considered that can inform the framework or its context?

By no means is this framework definitive, but it is one way to synthesize both the economic perspective [12] and the broader literature [5] with ethics [17]. Perhaps it could be used as a checklist to help generate discussion in committees or guide leaders tasked with making decisions surrounding policies on drug shortage allocations.

## 5. Using This New Framework for Thinking About How to Move Research and Policy Forward

Much of the discussion surrounds how policies should address the “supply chain” [19] aspects of the drug shortage problem (e.g., manufacturing incentives). This combined framework might be used to address issues at various points (from the macro to the micro levels—the broader supply chain of pharmaceuticals to the decision making of one pharmacist) that are all important parts of the pipeline that composes the drug shortage issue.

### 5.1. Long-Ingrained Incentives and the Overwhelming Role of Money

The issue of money is a huge underlying issue. Most drug shortages are in generics, and at the level of manufacturing companies, such companies are simply not incentivized to produce generics because of tiny profit margins [20]. The role of the free market induces competition that increases innovation and lowers the cost to the consumer, but the free market may also introduce the unintended consequence of patient harm because of cost incentives at various points in the supply chain. For example, pharmacy benefit managers (PBMs) act as middlemen, often making money by driving up drug prices [21]. Money also matters when one is using cost–benefit or return-on-investment analysis when considering policies. If cost is always the driver for the companies making the drugs, for the PBMs who take a cut, and even for those policymakers and researchers making the decisions, emphasizing or weighting more of the ethical dimensions related to patient harm due to drug shortages could be useful. For example, most people would likely agree that if drug shortages impact children with cancer, cost might become less of a factor than doing the right thing [7], and this would align with the “mitigating disadvantage” value of the ethical framework, but it also might conflict somewhat with the “equal moral concern” value (see Table 1).

### 5.2. Variability in Transparency and Consistency of Guidelines for Decision Making

Much of the discussion surrounding the supply chain on drug manufacturing focuses on transparency, ensuring that manufacturers, PBMs, and other players are being transparent about what they are doing [2]. The ideas and assumptions behind this transparency or accountability approach are to ensure that if everyone is openly reporting then the government agencies can adequately regulate the companies and to help solve the drug shortage issue. Of course, regulation can also cause unintended consequences or additional problems (e.g., see [4] about the drug shortage in ADHD medications). However, transparency at the level of using consistent guiding decision-making frameworks in allocating scarce medical resources is also an important issue. Fox et al. ([22], p. 335) note that even though most hospitals have a general policy on deciding who receives medications when there are shortages, it turns out that there is not much transparency when it comes down to who ultimately decides “which patients will receive medications that are in short supply” and that who decides “varies widely and is rarely guided by careful, transparent, and inclusive deliberation”.

### 5.3. Unclear Assessment on the Wide Range of Downstream Negative Impacts of Shortages

One of the key highlights of the scoping review [5] is that each medical subspecialty is focused on the negative impacts on their area, but perhaps does not fully consider the negative impacts in other subspecialties. Tucker et al. [5] also note there are few studies specifically taking the economics approach to isolate causes and that there is also a lack of studies seeking to adequately measure or quantify the degree of patient harm created by drug shortages in different domains and on various dimensions. This would address the “Does the policy anticipate and limit potential unintended consequences?” question. Finally, they note that more research should be directly focused on issues of ensuring marginalized groups are not negatively impacted, so addressing the “Mitigating disadvantage (equity)” dimension of the ethical framework found in Table 1.

### 5.4. Inequality of Journal rPestige and Media Coverage May Impact Equity across Fields and Policies or Research That Is Conducted

Chan [6] argues that media coverage is largely focused on high-profile journals and thus the topics that are published in those journals related to drug shortages (often on cancer-related drugs) become the policy problems that actually draw enough public attention to be solved. However, the author points out that iatrogenic malnutrition may potentially be an even larger problem, harming patients in different ways. This illustrates that decisions made by major journals on what topics they publish on, or which authors have connections to editors, and more broadly peer review, all are in part ethical decisions that have real (and potentially harmful intended or unintended) downstream consequences. What editors of mainstream news sites choose to cover also correspondingly has similar ethical conundrums.

### 5.5. Variability in Which Drugs Are Facing Shortages

One historically ongoing issue is that though there is some relative consistency regarding the types of drugs that commonly face shortages, this changes from year to year and so it is challenging for health systems to plan accordingly [23]. The ASHP and University of Utah drug shortages database [3] does provide some transparency on what drugs are currently facing a shortage, but this information is reported only after shortages have begun impacting hospitals and patients in various contexts well before.

### 5.6. Hospital Decisions to Make Up for the Drug Shortages

Connected to the inability to anticipate which drug shortages will occur is the need for hospitals to make up for unpredictable shortages in various ways which have other downstream impacts. Those solving the patient care issues are always showing that they have in some ways become accustomed to these challenges, and doctors cannot practice medicine freely as they have to make decisions and put patients on alternative—perhaps less efficacious—therapies. A specific hospital will likely create restriction criteria, and this is where the combined framework may help in the decision-making process. For example, medications may be prioritized for pregnant patients because they may not be able to take an alternative medication, but this may mean an elderly patient may not receive the optimal treatment. Ethically, it remains unclear how and in what ways one might decide to prioritize one life over another. Other consequences that have ethical issues are that larger hospitals tend to hoard drugs, meaning smaller community hospitals, often serving marginalized populations, may not be able to obtain drugs, or be able to make the trades with or be able to buy drugs from larger hospitals. These smaller hospitals may lose money as they might use more expensive alternative drug therapies, impacting their drug budget and thus increasing costs. This may lead to smaller hospitals not being able to stay afloat, thus being bought out by larger health systems.

### 5.7. Using the Ethical Framework as a Lens through Which to Consider Other Factors

In the multidisciplinary ethical framework for allocating scarce medical resources noted above, the first four questions are about the economic approach, which is largely quantitative in nature, focused on causes, and largely about money or cost. However, what if instead of thinking about costs or return on investment as the first consideration, thinking about how ethics (as outlined in Table 1; [17]) might be the driving factor for moving research and policy forward regarding drug shortages? Perhaps, if ethics were a core part of frameworks for decision making when seeking to reform all aspects impacting drug shortages, while also understanding that there are real issues of cost and other difficult-to-move structural issues, this might improve things for more patients.

## 6. Conclusions

Combining the economic perspective with the wider literature on drug shortages from various areas of pharmacy and medicine with a shared ethical framework may be a method to making decisions. This multidisciplinary ethical framework for allocating scarce medical resources might be useful when seeking to consider how to move both research and policy forward. For example, perhaps more research needs to be conducted in the area of the ethics of decision making related to drug shortages, or more work on the downstream consequences and impact on patients in often unintended ways would be useful. More importantly, when thinking about policy decisions that are often made largely on the basis of cost and incentives in the free market combined with structural issues with government regulations (i.e., the economic framework), perhaps using a framework including ethics could help policymakers make decisions that really prioritize patient interests, especially those who are marginalized, rather than the interests of companies and the bottom line.

## Figures and Tables

**Table 1:** Fundamental values for allocating scarce medical resources.

Maximizing benefit and reducing harm	“Preferential allocation of medical resources towards individuals who can gain most benefit and protection against harm; harms can be broad to include both health (e.g., death) and non-health (e.g., poverty); harms can occur directly from the disease and indirectly when health-care system functioning is compromised.”
Mitigating disadvantage (equity)	“Preferential allocation of medical resources towards people who are disadvantaged by income, race, ethnicity, religion, or other characteristics.”
Equal moral concern	“Treating similar people similarly, and not discriminating on the basis of morally irrelevant characteristics such as race, ethnicity, or religion; typically requires not treating people the same, but treating people in different circumstances (e.g., in communities with a higher or lower burden of COVID-19) differently.”
Reciprocity	“Preferential allocation of medical resources towards people, communities, or countries who in the past took on burdens to address the current health problem.”
Instrumental value	“Preferential allocation of medical resources towards people who will be able to mitigate harms and disadvantage of others; not an independent value but facilitates realizing the other values particularly benefiting people.”

Note. Adapted from Table 2 of Emanuel and Persad [17]. Text is in quotes as it is taken from that table to ensure accuracy, but is slightly copyedited to match U.S.-focused language formatting.

## Data Availability

Not applicable.
